# Frailty shows enhanced predictive capacity for 1-year unplanned readmission comparing to reason for admission

**DOI:** 10.1093/geroni/igaf122.2371

**Published:** 2025-12-31

**Authors:** Junpeng Kan, Ying Sun, Wen Tang, Yunli Xing, Qing Ma

**Affiliations:** Beijing Friendship Hospital, Capital Medical University, Beijing, Beijing, China (People’s Republic); Beijing Friendship Hospital, Capital Medical University, Beijing, Beijing, China (People’s Republic); Beijing Friendship Hospital, Capital Medical University, Beijing, Beijing, China (People’s Republic); Beijing Friendship Hospital, Capital Medical University, Beijing, Beijing, China (People’s Republic); Beijing Friendship Hospital, Capital Medical University, Beijing, Beijing, China (People’s Republic)

## Abstract

**Background:**

Frailty, a prevalent geriatric syndrome, predisposes older adults to adverse outcomes including disability and premature mortality. Unplanned hospital readmissions impose substantial economic burdens on families and challenge healthcare systems.

**Objective:**

To investigate the impact of frailty on 1-year unplanned readmissions among elderly inpatients and further analyze its predictive utility across subgroups stratified by admission causes (acute vs. chronic conditions).

**Methods:**

This prospective cohort study enrolled patients aged ≥65 years from the Geriatrics Department of Beijing Friendship Hospital, Capital Medical University. Frailty status was assessed using the FRAIL scale, categorizing participants into non-frail and frail groups. Participants were stratified by admission diagnosis (acute/chronic conditions). Baseline demographics, medical histories, and laboratory parameters were collected. Electronic medical records were reviewed to identify unplanned readmissions within 1 year.

**Results:**

Among 414 enrolled patients. Median age was 82 years (IQR 72∼88), 58.9% (n = 244) were frail. During the 1-year follow-up period, 173 patients (42.8%) experienced unplanned readmissions. Frailty independently predicted 1-year unplanned readmissions (OR = 1.694, 95% CI:1.013–2.830, P = 0.044), with a significant interaction between frailty and admission cause (P for interction 0.006). Subgroup analysis revealed enhanced predictive accuracy of frailty comparing to chronic disease-related admissions.

**Conclusions:**

Frailty serves as an independent risk factor for 1-year unplanned readmissions in elderly inpatients, particularly among those hospitalized for chronic conditions. Systematic frailty screening at admission coupled with tailored physical rehabilitation programs may mitigate readmission risks.

